# Characterization of the preweaned Holstein calf fecal microbiota prior to, during, and following resolution of uncomplicated gastrointestinal disease

**DOI:** 10.3389/fmicb.2024.1388489

**Published:** 2024-05-24

**Authors:** Rachel A. Claus-Walker, Giovana S. Slanzon, Lily A. Elder, Holly R. Hinnant, Chris M. Mandella, Lindsay M. Parrish, Sophie C. Trombetta, Craig S. McConnel

**Affiliations:** Department of Veterinary Clinical Sciences, Field Disease Investigation Unit, Washington State University, Pullman, WA, United States

**Keywords:** calf diarrhea, gastrointestinal microbiota, gut health, case definitions, microbiome

## Abstract

Little is known about shifts in the fecal microbiome of dairy calves preceding and following the incidence of gastrointestinal disease. The objective of this cohort study was to describe the fecal microbiome of preweaned dairy calves before, during, and after gastrointestinal disease. A total of 111 Holstein dairy calves were enrolled on 2 dairies (D1 and D2) and followed until 5 weeks old. Health assessments were performed weekly and fecal samples were collected every other week. Of the 111 calves, 12 calves from D1 and 12 calves from D2 were retrospectively defined as healthy, and 7 calves from D1 and 11 calves from D2 were defined as diarrheic. Samples from these calves were sequenced targeting the 16S rRNA gene and compared based on health status within age groups and farms: healthy (0–1 week old) vs. pre-diarrheic (0–1 week old), healthy (2–3 weeks old) vs. diarrheic (2–3 weeks old), and healthy (4–5 weeks old) vs. post-diarrheic (4–5 weeks old) calves. Healthy and diarrheic samples clustered together based on age rather than health status on both farms. Based on linear discriminant analysis, a few species were identified to be differently enriched when comparing health status within age groups and farm. Among them, *Bifidobacterium* sp. was differently enriched in pre-diarrheic calves at D1 (0–1 week old) whereas healthy calves of the same age group and farm showed a higher abundance of *Escherichia coli*. *Bifidobacterium* sp. was identified as a biomarker of fecal samples from healthy calves (2–3 weeks old) on D1 when compared with diarrheic calves of the same age group and farm. Feces from diarrheic calves on D2 (2–3 weeks old) were characterized by taxa from *Peptostreptococcus* and *Anaerovibrio* genera whereas fecal samples of age-matched healthy calves were characterized by *Collinsella aerofaciens* and *Bifidobacterium longum*. After resolution of uncomplicated diarrhea (4–5 weeks old), *Collinsella aerofaciens* was more abundant in D2 calves whereas *Bacteriodes uniformis* was more abundant in D1 calves. Taken together, these findings suggest that the age of the preweaned calf is the major driver of changes to fecal microbiome composition and diversity even in the face of uncomplicated gastrointestinal disease.

## 1 Introduction

Bacteria residing in the intestines of vertebrates support a unique ecosystem which impacts the entirety of the animal. For example, the microbiome of the mammalian gut provides multiple benefits to the health and function of the gut including impacts to mucosal architecture (Sharma et al., 1995), allergies (Round et al., 2011), and immune cell differentiation (Smith and Garrett, 2011). In Holstein calves, evidence exists to suggest transcriptome changes involving immune development occur in association with microbial colonization (Liang et al., 2014). However, these benefits can be disrupted by occurrences of early life gastrointestinal (GI) disease and associated antimicrobial usage (Arrieta et al., 2014; Zeissig and Blumberg, 2014; Schwartz et al., 2020).

Although much of the current microbiome research is partial to human health (Waldor et al., 2015), microbial community and population changes in pre-weaned dairy calves with GI disease have been documented (Gomez et al., 2017; Kim et al., 2021). In fact, the most important health problem in neonatal calves is GI disease manifested as diarrhea (Torsein et al., 2011), and accounts for the majority of preweaned heifer deaths (USDA, 2014). Diarrhea is a multifactorial disease with numerous infectious and noninfectious risk factors associated with parturitional events, pathogen exposure, environmental conditions, nutritional state, immune status, breed, and stocking density (Bendali et al., 1999; Svensson et al., 2003; Klein-Jöbstl et al., 2014). One intrinsic calf factor affecting diarrhea risk is the evolution and maturation of the calf’s GI tract. From birth to weaning the neonatal calf GI tract undergoes rapid microbial and structural evolution and disruptions of this evolution are associated with GI disease in young calves (Meale et al., 2017). It takes 3 to 4 weeks after birth for the GI bacterial community structure to reach a certain degree of stabilization (Abecia et al., 2014; Rey et al., 2014). This period appears to be critical for the colonization of the GI microbiota but the persistency of the microbial imprint is unknown and in need of further clarification (Yáñez-Ruiz et al., 2015). Major changes in GI tract anatomy and the associated microbiota occur as calves age and progress from milk feeding to the development of a functional rumen (Oikonomou et al., 2013). Although certain components of the microbiota are always present in large, and approximately constant numbers in the healthy gut, alterations of the fecal microbiota have been associated with diseases and antimicrobial treatments (Oikonomou et al., 2013; Oultram et al., 2015; Weese and Jelinski, 2017; Zeineldin et al., 2018). Consequently, fecal microbiota structure and function need investigated within the dairy farm field setting in an effort to more fully support calf health management and antimicrobial stewardship related to microbiota perturbations (Mahendran, 2020).

Microbiota community dynamics can be understood through the employment of ecological monitoring tools (Gilbert and Lynch, 2019). Communities are assemblages of species and serve as the fundamental unit of ecological organization (Christian et al., 2015). Many healthy GI communities in neonatal dairy calves have been shown to be abundant in Firmicutes and Bacteriodetes phyla (Malmuthuge et al., 2014; Alipour et al., 2018; Hennessy et al., 2021). These communities can be affected during GI disease leading to a perturbation of assemblages (Gomez et al., 2017; Kim et al., 2021). Assemblages of diarrheic calves have demonstrated an abundance of Firmicutes, Bacteroidetes (Malmuthuge et al., 2014; Alipour et al., 2018; Hennessy et al., 2021), Proteobacteria (Gomez et al., 2017), and Verrucomicrobia (Kim et al., 2021) phyla. Much of this research is limited to the description of dysbiosis during GI diseases.

A recent review suggested that in the future, routine diagnostic methods will include understanding and monitoring calf metabolomes and GI microbiomes as they are essential input for maintaining health and productivity in dairy herds (Britt et al., 2018). The importance of understanding GI microbiomes and phenotypes also is bolstered by evidence that the neonatal microbiota works through immunologic and inflammatory mechanisms to influence the course of disease (Belkaid and Hand, 2014). In fact, studies are beginning to explore the connection between GI and respiratory systems (Zhang et al., 2020), and a new approach through fecal microbiome analysis is needed to understand the influence of a microbial community on calf health and disease progression and resolution. With this in mind, the objective of the current study was to describe the fecal microbiota in preweaned Holstein heifer calves before, during, and after GI disease as evidenced by diarrheal events. We hypothesized that microbial diversity, abundance, and composition would differ based on age and the presence or absence of GI disease progression.

## 2 Materials and methods

### 2.1 Ethics statement

The research protocol was reviewed and approved by the Institutional Animal Care and Use Committee of Washington State University (IACUC protocol #6859).

### 2.2 Animals and study design

This cohort study was conducted on 2 commercial dairies (D1 and D2) in the state of Washington during 2020 and 2021. Study farms were selected based on established relationships with the Field Disease Investigation Unit (FDIU) at Washington State University’s College of Veterinary Medicine. Farm D1 had an average lactating cow inventory of approximately 2,250 cattle, whereas D2 averaged approximately 11,000 lactating cattle. A convenience sample of 25–30 Holstein-Friesen calves between 1 and 11 days of age (less than 2 weeks old) were enrolled into cohorts during 3 discrete 12-week sampling periods (November 2020–January 2021; February–April 2021; May–August 2021). A total of 111 calves were followed for this project. Twenty-five calves were enrolled on D2 during November 2020, and followed through January 2021. Twenty-six calves were enrolled on D1 during February 2021, and followed through April 2021. Thirty calves on each dairy were enrolled during May 2021, and followed through the end of July or early August 2021. All primary care, including treatments and husbandry, were performed by on-farm personnel who received *ad-hoc*, on-the-job training to assess calf health. Workers utilized their experience, collaboration with veterinarians, and corroborative health observations such as posture, stool consistency, risk age, appetite, and attitude to make treatment decisions.

On both farms calves received 3.8 liters (1 gallon) of (pasteurized, D1; unpasteurized, D2) colostrum (Brix refractometer ≥22%) via oral intubation within 30 min of birth and a second gallon (Brix refractometer <22%) 8–12 h later. On D1 calves were bucket fed 3 quarts (2.8 liters) twice daily of pasteurized hospital and bulk tank milk mixed with milk replacer to a achieve a total solids of 14%. Weaning was abrupt at 60 days of age. Calf grain and alfalfa pellets were available from 1 day of age with grain fed to a maximum of 1.4 kg (3.0 lbs) and alfalfa pellets fed *ad libitum*. On D2 calves were bucket fed pasteurized hospital milk and unpasteurized bulk tank milk mixed with milk replacer as needed to achieve a total solids of 13–13.5%. Calves were provided 3 quarts (2.8 liters) of the milk mixture twice a day from 1 to 20 days of age, 2.5 quarts (2.4 liters) twice a day from 21 to 39 days of age, and 2 quarts (1.9 liters) twice a day from 40 to 55 days of age at which point they were weaned. A total mixed ration of calf pellets, rolled corn, molasses, and small percentage of chopped alfalfa was offered from day 1 of age. On D1 all calves received a single, intranasal dose of Nasalgen 3 (Merck Animal Health, Kenilworth, NJ) at approximately 2 weeks of age. On D2 all calves received a single, intranasal dose of Inforce 3 (Zoetis Animal Health, Parsippany, NJ) at approximately 4 weeks of age.

### 2.3 Health assessments and interventions

Health status of each calf was assessed on the day of enrollment and then every other week (November 2020–January 2021; February–April 2021), or weekly (May–August 2021), based on a standardized calf health-scoring chart (McGuirk, 2008). Research personnel were trained to perform health assessments by the project’s PI (McConnel). Health scores were based on the evaluation of rectal temperature, nasal discharge, coughing, ocular discharge, fecal consistency, distal limb joints, ears, and the umbilicus. Rectal temperature was scored as follows: 0 = 37.8–38.3°C (100.0–100.9°F), 1 = 38.3–38.8°C (101.0–101.9°F), 2 = 38.9–39.4°C (102.0–102.9°F), and 3 ≥ 39.4°C (≥ 103.0°F). Nasal discharge was scored as follows: 0 = no discharge, 1 = small amount of unilateral cloudy discharge, 2 = bilateral cloudy or excessive mucous discharge, and 3 = copious bilateral mucopurulent discharge. Cough scores were based on the following: 0 = no cough, 1 = induced single cough, 2 = induced repeated or occasional spontaneous coughs, and 3 = repeated spontaneous coughs. Ocular discharge was scored as follows: 0 = no discharge, 1 = small amount of ocular discharge, 2 = moderate amount of bilateral discharge, and 3 = heavy ocular discharge. Fecal consistency was scored based on the following criteria: 0 = well-formed, 1 = semi-formed and pasty, 2 = loose, but did not run off a gloved hand, and 3 = watery, easily ran off a gloved hand. Joint scores were based on the following designations: 0 = no heat, swelling or pain, 1 = slight swelling, not warm or painful, 2 = swelling with pain or heat, slight lameness, and 3 = swelling with severe pain, heat, and lameness. Ear position was observed and scored as follows: 0 = no abnormal movement or positioning, 1 = ear flicking, 2 = slight unilateral ear droop, and 3 = severe head tilt or bilateral ear droop. Finally, the umbilicus was palpated weekly and scored as follows: 0 = no heat, swelling or pain, 1 = slightly enlarged, not warm or painful, 2 = slightly enlarged with slight pain or moisture, and 3 = enlarged with pain, heat, or malodorous discharge. In addition, a behavioral assessment was conducted for calves that either were not bright, alert and responsive or had clinical scores ≥2 in any of the above parameters. Behavioral scores were based on assessments of demeanor, ear position, mobility, interaction, suckling reflex, scleral injection, and ocular recession for a total possible behavioral score of 28 (Supplementary Table S1).

Therapeutic interventions on each farm were performed by farm personnel and subsequently recorded in a Dairy Comp database (Valley Agricultural Software, Tulare, CA). Therapeutic interventions included individual treatments or a combination of the following: IV fluid therapy (Lactated Ringer’s solution), oral electrolytes, laxatives, anti-inflammatories (flunixin meglumine or dexamethasone), or variable IM, IV, or SQ antibiotics (ceftiofur, florfenicol, penicillin, enrofloxacin, tulathromycin, ampicillin, or oxytetracycline). Calves receiving antimicrobial, laxative, or anti-inflammatory treatment prior to or at the time of sample collection were excluded from microbiome analysis.

### 2.4 Group assignment

For the purposes of microbiome evaluation, calves were retrospectively placed into 2 groups within each farm: healthy and diarrheic (scours uncomplicated by comorbidities). Healthy calves were continually free of pyrexia, respiratory disease, abnormal behavioral scores, and diarrhea during their health assessments throughout the first 5 weeks of life. Pyrexia was based on a score of 3, which indicated a temperature ≥ 39.4°C (≥ 103.0°F). Respiratory disease was based on a score of ≥2 for the nasal, cough, eye, or ear categories. Finally, a fecal score of ≥2 excluded calves from the healthy group.

Calves in the diarrheic group were selected based on the presence of diarrhea (fecal score of ≥2) at 2–3 weeks old with an absence of clinical disease 2 weeks before and after the diarrheal event, indicating complete clinical resolution of diarrhea. The diarrheal time period was predicated on the majority of scours in these cohorts occurring in calves that were 2–3 weeks old. Animals with pyrexia, signs of respiratory disease, or treatment with laxatives, anti-inflammatories, or antimicrobials ultimately were excluded from analysis. If a calf presented with abnormal behavioral scores outside of the designated diarrheal interval of 2–3 weeks old, it was also excluded from this group.

In summary, the health status of calves enrolled into groups was strictly defined in this study as healthy or diarrheic based on a combination of all the health parameters assessed, disease incidence, behavior changes, and interventions. The summary of the measurements that define each calf health status and age group can be found in the Supplementary Table S2. Based on the health status of the diarrheic group, we compared samples from healthy (0–1 week old) vs. pre-diarrheic (0–1 week old), healthy (2–3 weeks old) vs. diarrheic (2–3 weeks old), and healthy (4–5 weeks old) vs. post-diarrheic (4–5 weeks old) calves for microbiome analysis purposes.

### 2.5 Fecal sample collection

Fecal samples were obtained every other week from rectum from all calves in each cohort (150–200 g). Samples were immediately labeled and stored on ice for transport back to the Field Disease Investigation Lab at Washington State University, Pullman WA, where they were then stored at −80°C until preparation for sequencing. At the study’s conclusion in August 2021, fecal samples were slightly thawed, mixed, and 1 g of feces was transferred to DNA/RNA Shield Fecal Collection Tubes (Zymo Research, Irvine, CA). The preserved feces were then sent to Zymo Research for DNA extraction and 16S-rRNA V3-V4 region amplification and sequencing.

### 2.6 Amplification and sequencing of bacterial 16S rRNA gene

The ZymoBIOMICS Targeted Metagenomic Sequencing service (Zymo Research, Irvine, CA) was utilized for bacterial 16S rRNA gene amplification and sequencing of the V3-V4 region (primers 341F-806R) using an Illumina MiSeq with a v3 reagent kit (600 cycles).

### 2.7 Bioinformatic and statistical analyses

All data processing and taxonomy assignment was conducted by Zymo Research (Irvine, CA). Unique amplicon sequences were inferred from raw reads using the Dada2 pipeline (Callahan et al., 2016). Chimeric sequences were also removed with the Dada2 pipeline. Uclust from Qiime v.1.9.1 was used to perform taxonomy assignment (Caporaso et al., 2010). Taxonomy was assigned with the Zymo Research Database (Zymo Research, Irvine, CA) as a reference. ASV abundance table, taxonomy table, and read processing summary table are provided in Supplementary Table S3. All detected ASVs were included in the analysis. The initial analysis only compared calves within a farm. For example, microbiome analyses of calves in D1 cohorts were completed separately from D2 calves. The independent analysis was deemed necessary due to varying management practices on each farm. Relative abundance data was calculated based on the number of sequences reads and total reads per sample. Alpha diversity was measured using the *vegan* (Dixon, 2003) package in R program using the observed species, Shannon, and Simpson index metrics. Differences between alpha diversity indexes across groups (health status and age) and farms (D1 and D2) were calculated by Analysis of Variance (ANOVA). Afterwards, post-hoc analysis was done using Tukey’s Honestly Significant Difference (HSD) using the *agricolae* package. A non-parametric approach using Kruskal-Wallis test and pairwise comparisons using the Wilcoxon rank sum test with correction for multiple testing from R’s package *stats* were also conducted to assess differences between alpha diversity indexes across groups. Beta diversity analysis was calculated using the ordinate function in R’s *phyloseq* package (McMurdie and Holmes, 2013) to create a principal coordinates analysis (PCoA) based on the Bray-Curtis distance. To understand dissimilarities of healthy calves between farms, beta-diversity was performed on samples from healthy calves from both dairies. In addition, beta diversity analysis was used to compare samples from calves with different health status within dairies. A permutational multivariate ANOVA (PERMANOVA) test was implemented with the *vegan* package in R (Dixon, 2003). Differential abundance of bacterial taxa between study groups was analyzed through linear discriminant analysis effect size (LEfSe) bar plots. Linear discriminant analysis (LDA) scores provided by Zymo Research (Irvine, CA) indicates bacteria that have significant different abundances among different groups (LDA score > 2 and *p*-value <0.05 criteria; Segata et al., 2011).

## 3 Results

### 3.1 Sample population

A total of 111 calves were enrolled in this study from November 2020 to August 2021. Of the 111 calves, 12 calves from D1 and 12 calves from D2 met the qualifications to be considered part of the healthy group (Supplementary Table S2). Additionally, 7 calves from D1 and 11 calves from D2 were determined to fit the diarrheic group classification. Fecal samples were collected from each of the 42 calves at 3 timepoints during the ages of 0–1, 2–3, and 4–5 weeks old. The diarrheic group only had diarrhea during the 2–3 weeks of age collection (i.e., they were free of clinical disease during the ages of 0–1 and 4–5 weeks; Supplementary Table S2). The dataset of all 126 samples were analyzed.

### 3.2 Alpha diversity analysis

**Figure 1:**
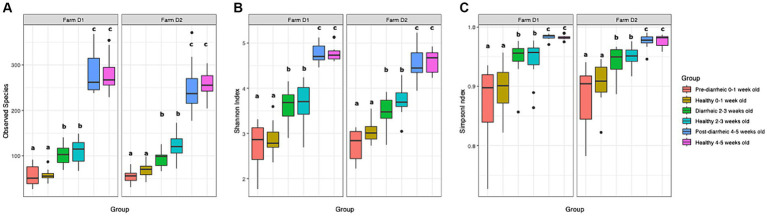
Observed species index **(A)**, Shannon index **(B)**, and Simpson index **(C)** are plotted for samples grouped by age and health status. The median is depicted by the line inside the box whereas the lowest and highest values observed are represented by the whiskers.

A total of 2,895 ASVs were identified and analyzed in our dataset. The mean relative abundance of the most abundant ASVs across all samples are shown in Supplementary Figure S1. In order to identify changes in diversity across groups, we used ANOVA to compare fecal microbial diversity by farm (D1, D2) and health status (healthy, pre-diarrheic, diarrheic, post-diarrheic) grouped by week of age (0–1, 2–3, 4–5). The observed species, Shannon, and Simpson indices increased as calves aged in both healthy and diarrheic groups within each farm (Figure 1). All 3 indices indicated differences in richness and evenness over time (*p* < 0.001), but no differences between farms (*p* > 0.1). Tukey’s test results indicated no differences across health status within age groups. In addition, a non-parametric approach using Kruskal-Wallis test and pairwise comparisons using the Wilcoxon rank sum test showed similar results for all alpha diversity indexes analyzed, indicating no differences across health status within age groups (*p* > 0.05).

### 3.3 Beta diversity of healthy calves compared across farms and ages

**Figure 2:**
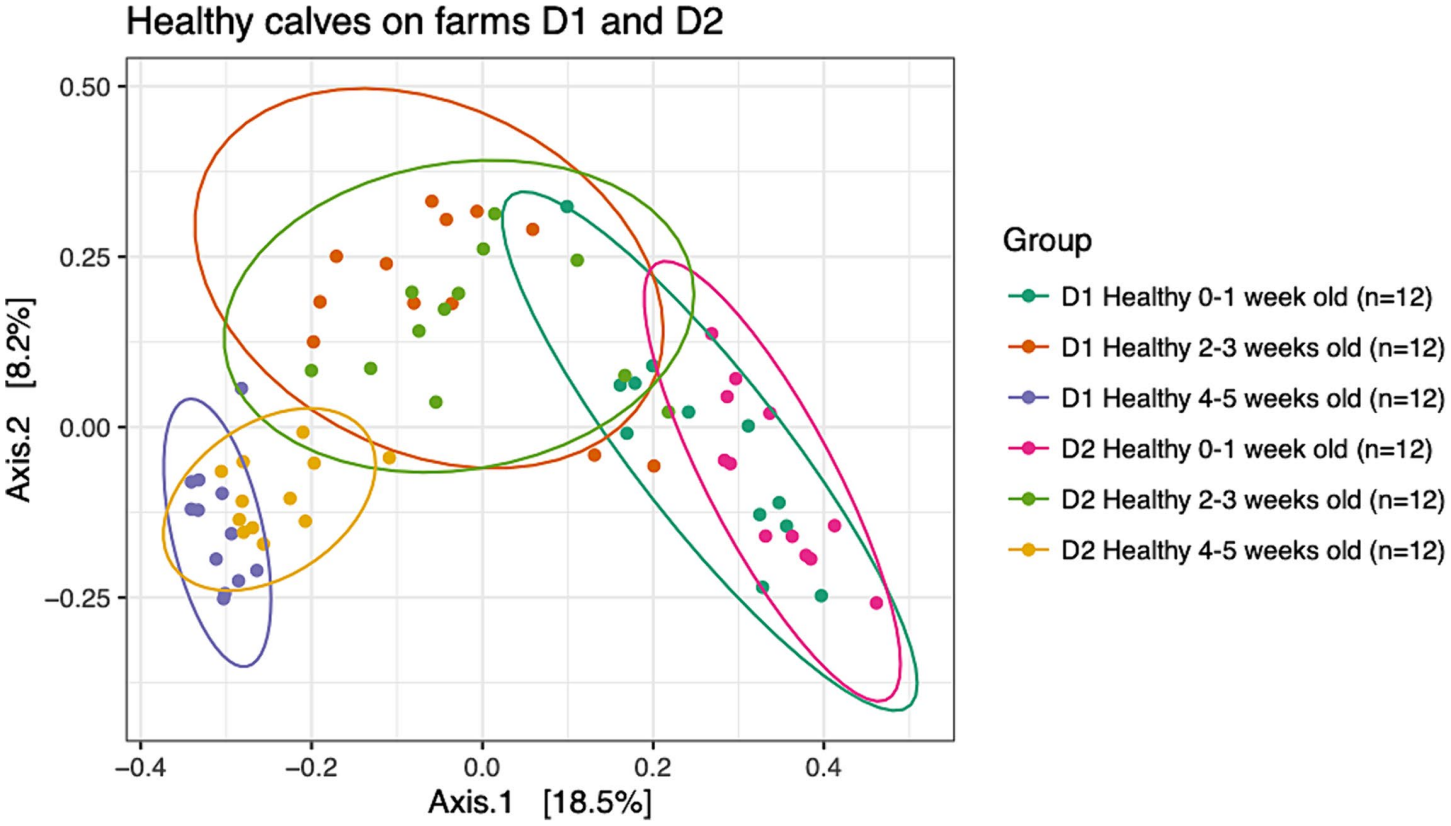
Principal coordinate analysis (PCoA) based on Bray-Curtis compositional dissimilarity of the bacterial 16S rRNA gene sequence data for fecal samples collected from healthy calves on farms D1 (*n* = 12) and D2 (*n* = 12) at 0–1, 2–3, and 4–5 weeks old. The proportion of variance explained by each principal coordinate axis is expressed by the corresponding axis label. Points represent individual samples. Samples that are more similar to one another appear closer together. The ellipses represent a 95% confidence interval calculated based on a t-distribution.

To understand dissimilarities of healthy calves between farms, beta-diversity analysis based on the Bray-Curtis metric was performed on samples from healthy calves from both dairies as depicted in Figure 2. PERMANOVA results showed that the fecal microbial communities were dissimilar based on farm (*R^2^* = 0.04; *p* < 0.001) and age (*R^2^* = 0.21; *p* < 0.001). However, the bacterial composition of fecal samples from age-matched animals on farms D1 and D2 clustered together highlighting the greater influence that age had on the calves’ fecal microbial community. More specifically, pairwise comparisons showed that the fecal microbial community of calves that were 0–1 week old differed from 2 to 3 weeks old calves (*p* = 0.003) and 4–5 weeks old calves (*p* = 0.003), and samples collected from calves that were 2–3 weeks old differed from 4 to 5 weeks old calves (*p* = 0.003).

### 3.4 Beta diversity of diarrheic and healthy calves on farms D1 and D2

**Figure 3:**
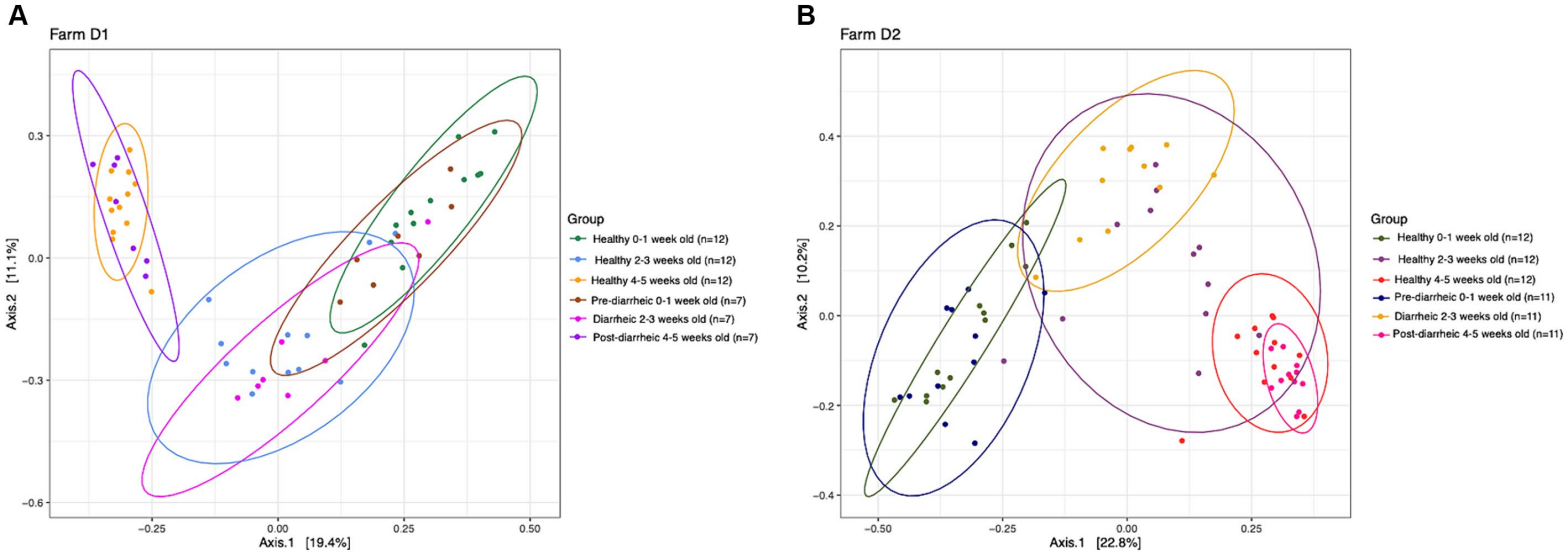
Principal coordinate analysis (PCoA) based on Bray-Curtis compositional dissimilarity of the bacterial 16S rRNA gene sequence data for fecal samples collected from healthy (*n* = 12) and diarrheic (*n* = 7) calves on farm D1 (panel **A**), and healthy (*n* = 12) and diarrheic (*n* = 11) calves on farm D2 (panel **B**) at 0–1, 2–3, and 4–5 weeks old. The proportion of variance explained by each principal coordinate axis is expressed by the corresponding axis label. Points represent individual samples. Samples that are more similar to one another appear closer together. The ellipses represent a 95% confidence interval calculated based on a t-distribution.

PERMANOVA analysis suggested that health status and week of age (D1: *R^2^* = 0.30, *p* = 0.001; D2: *R^2^* = 0.32, *p* = 0.001) impacted the community structure on both dairies. However, healthy and diarrheic samples appeared to cluster together based on age group rather than health status (Figure 3). The lack of separation between health status within an age group highlighted the more dominant impact of age on the fecal microbiota of calves. Pairwise comparisons showed no differences across health status (healthy vs. pre-, during, and post-diarrhea) within sampling times (*p* > 0.05), except for healthy and diarrheic calves that were 2–3 weeks old on D2 (*p* = 0.03). However, on each farm 0–1 week old calves within the pre-diarrheic group demonstrated differences in microbial diversity when compared to the older diarrheic and post-diarrheic groups (*p* < 0.05).

### 3.5 Differential abundance analysis of farm D1

**Figure 4:**
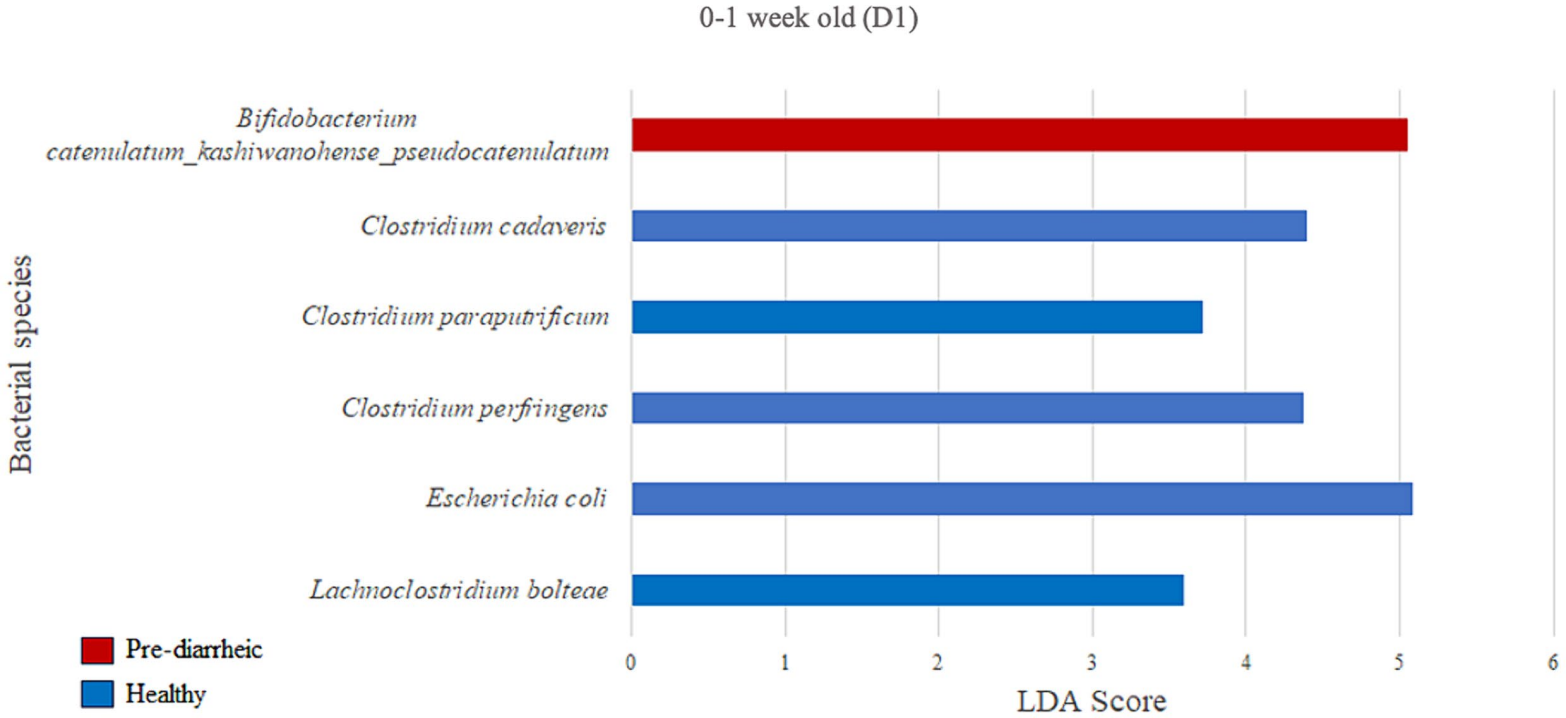
Linear discriminant analysis (LDA) effect size (LEfSe) bar plot comparing D1 pre-diarrheic calves (*n* = 7; red bars) with D1 healthy calves (*n* = 12; blue bars) at 0–1 week old. The LDA score (log_10_) indicates the effect size of each differentially abundant bacterial taxon (*p* < 0.05).

**Figure 5:**
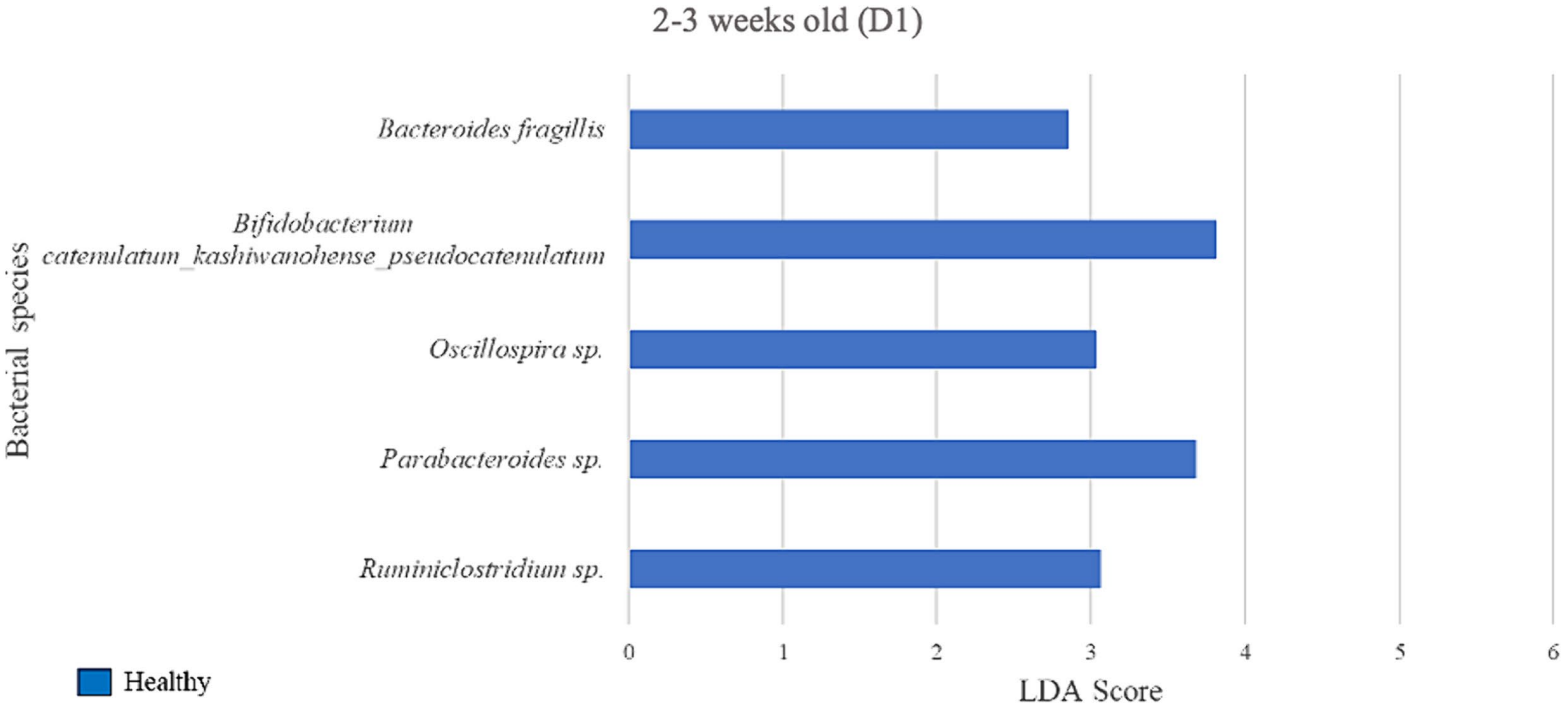
Linear discriminant analysis (LDA) effect size (LEfSe) bar plot comparing D1 diarrheic calves (*n* = 7) with D1 healthy calves (*n* = 12; blue bars) at 2–3 weeks old. The LDA score (log_10_) indicates the effect size of each differentially abundant bacterial taxon (*p* < 0.05).

**Figure 6:**
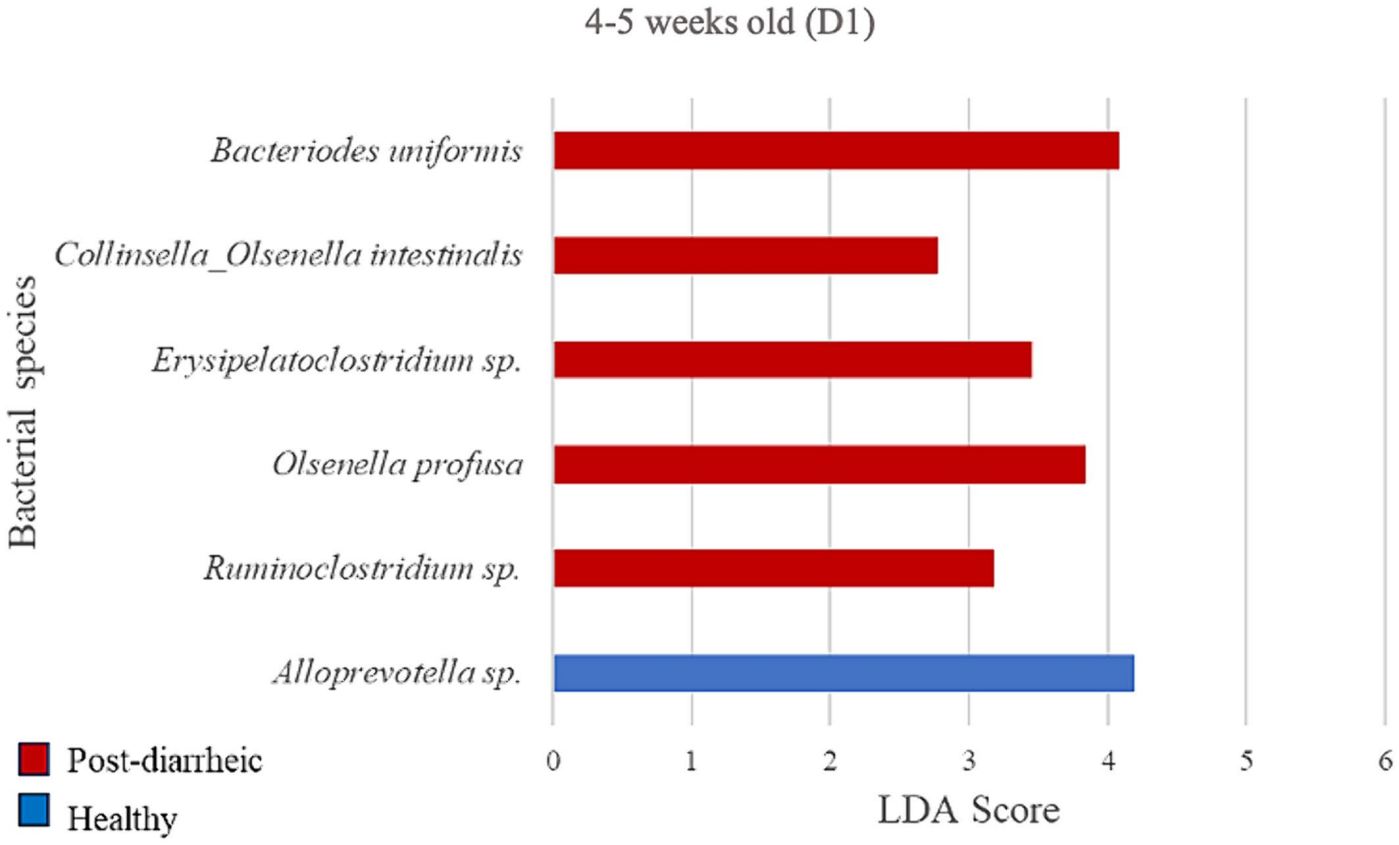
Linear discriminant analysis (LDA) effect size (LEfSe) bar plot comparing D1 post-diarrheic calves (*n* = 7; red bars) with D1 healthy calves (*n* = 12; blue bars) at 2–3 weeks old. The LDA score (log_10_) indicates the effect size of each differentially abundant bacterial taxon (*p* < 0.05).

We next assessed the differential abundance of microbial species in the feces of healthy vs. pre-diarrheic, diarrheic, and post-diarrheic calves on D1 (Figures 4–6). Paired with effect size measurements (LEfSe), linear discriminant analysis of the abundance data was used to generate bar graphs to display discriminatory taxa of each group. During 0–1 week old, the calf fecal microbiome of the pre-diarrheic group showed a higher abundance of *Bifidobacterium* sp. of the Actinobacteria phylum (LDA = 5.05) when compared with samples from healthy calves of the same age group. On the other hand, *Escherichia coli* of the Proteobacteria phylum and *Clostridium* spp. of the Firmicutes phylum were more abundant in healthy calves from the same age group (0–1 week old; LDA = 5.08 and 3.71, respectively; Figure 4).

During 2–3 weeks old, 5 bacterial species were identified as biomarkers of the fecal microbiome of the healthy group when compared with the diarrheic group on D1 (Figure 5), including *Bifidobacterium* spp. (LDA = 3.81) and an unclassified bacterium of the *Parabacteroides* genus (LDA = 3.68). The diarrheic group did not yield enriched species meeting the LDA score (> 2) and *p*-value (< 0.05) criteria.

At the final timepoint of 4–5 weeks old, the only bacterial species differently enriched in the healthy group on D1 was an unclassified organism of the *Alloprevotella* genus (LDA = 4.20; Figure 6). Additionally, 5 species were more abundant in the diarrheic group when compared with samples from healthy calves of the same age group, especially *Bacteroides uniformis* of the Bacteroidetes phylum (LDA = 4.09).

### 3.6 Differential abundance analysis of farm D2

**Figure 7:**
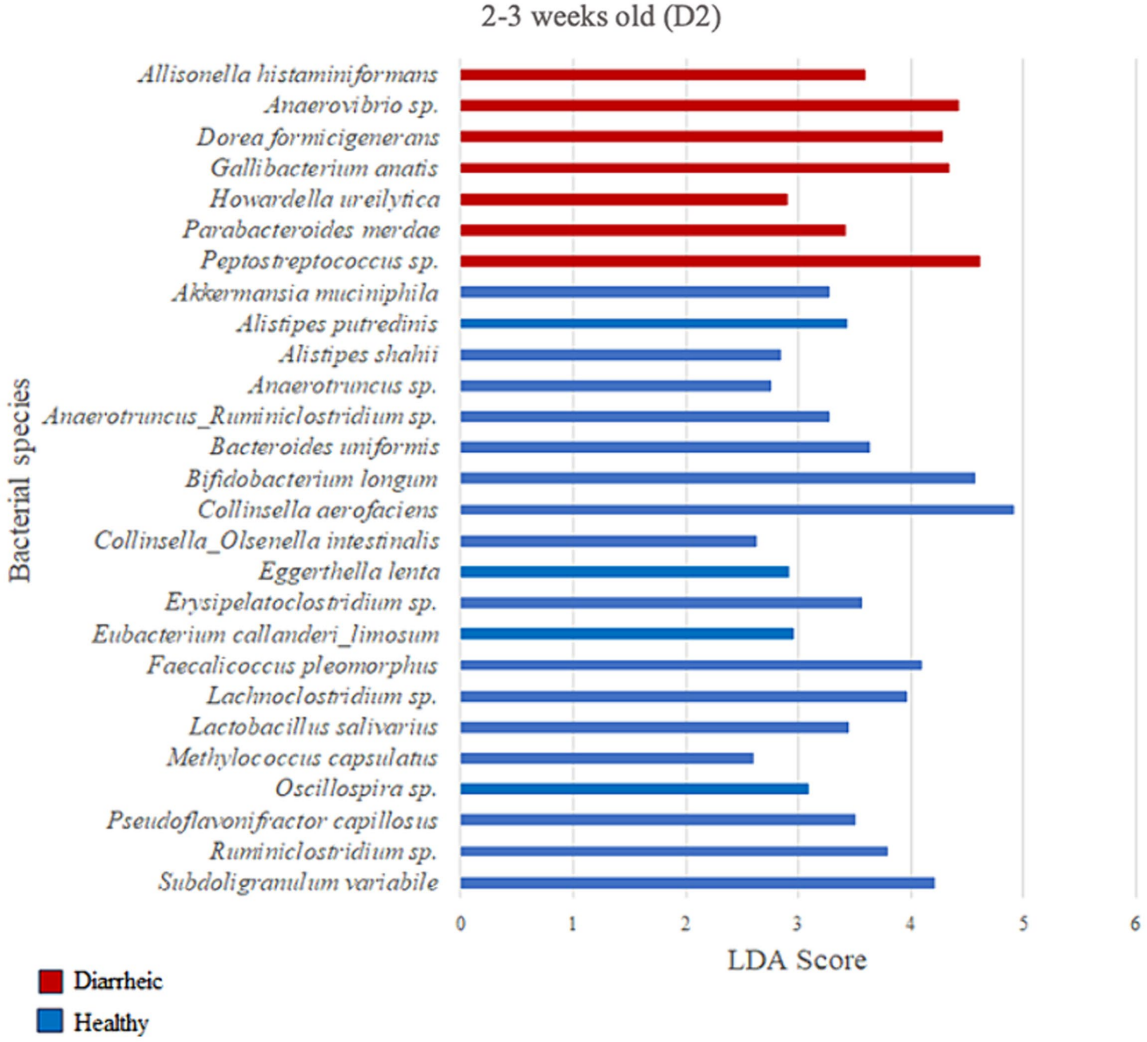
Linear discriminant analysis (LDA) effect size (LEfSe) bar plot comparing D2 diarrheic calves (*n* = 11; red bars) with D2 healthy calves (*n* = 12; blue bars) at 2–3 weeks old. The LDA score (log_10_) indicates the effect size of each differentially abundant bacterial taxon (*p* < 0.05).

**Figure 8:**
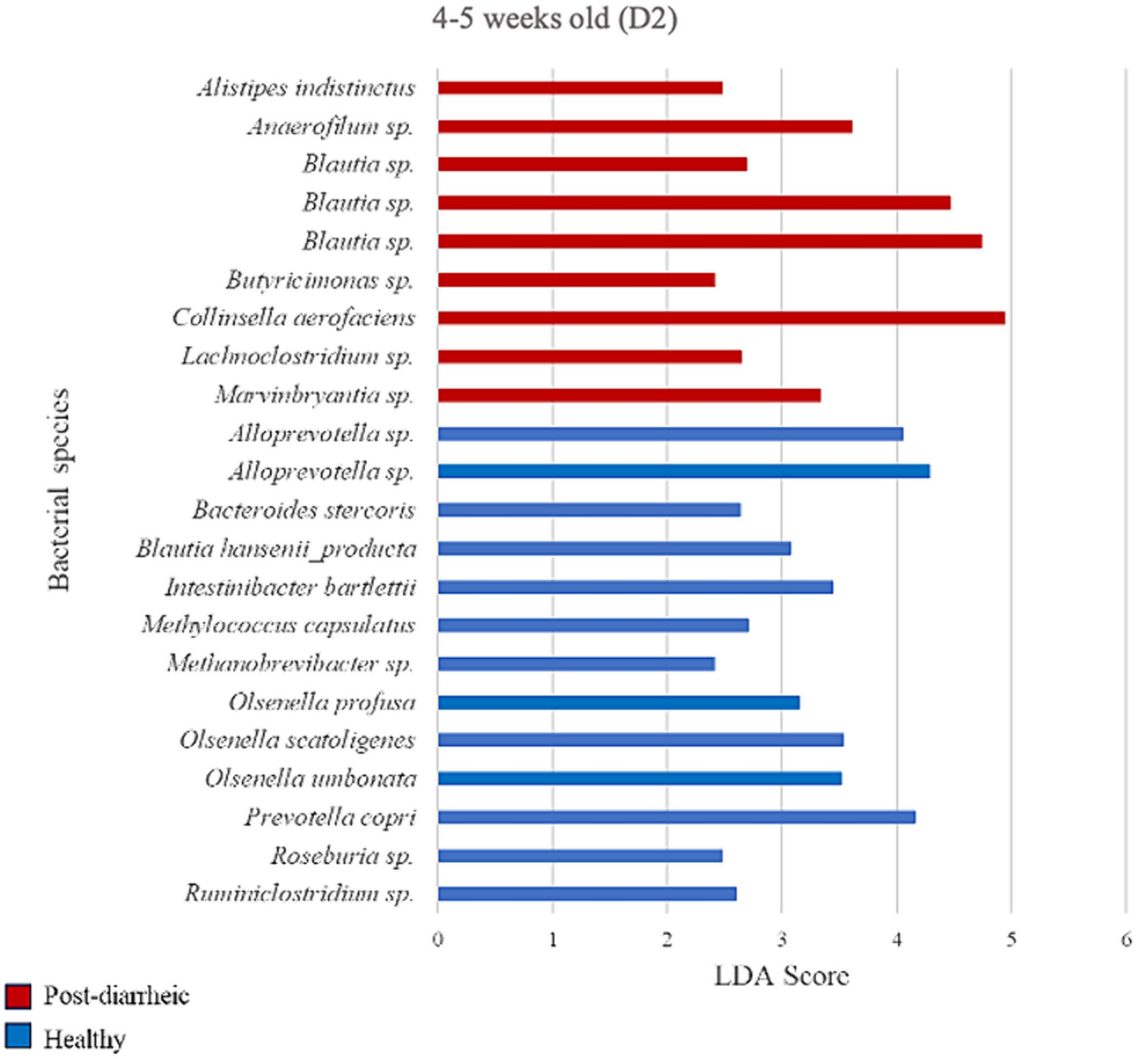
Linear discriminant analysis (LDA) effect size (LEfSe) bar plot comparing D2 post-diarrheic calves (*n* = 11; red bars) with D2 healthy calves (*n* = 12; blue bars) at 4–5 weeks old. The LDA score (log_10_) indicates the effect size of each differentially abundant bacterial taxon (*p* < 0.05).

Based on the LDA score (>2) and p-value (*p* < 0.05) criteria, the analysis comparing D2 calves within 0–1 week old did not yield multiple differently enriched species. However, *Methylococcus capsulatus* was more abundant in healthy animals (LDA = 2.69; *p* = 0.001) during that timeframe when compared with pre-diarrheic calves of the same age group. Numerous differently enriched taxa were present in the feces of the healthy and diarrheic groups on D2 at 2–3 and 4–5 weeks old (Figures 7, 8). At the 2–3 weeks old time point several species from the Firmicutes phylum characterized the diarrheic group. Specifically, bacteria from the *Peptostreptococcus* and *Anaerovibrio* genera (LDA > 4.43). The healthy group demonstrated higher abundance of species from both Firmicutes and Actinobacteria phyla during 2–3 weeks old, with *Collinsella aerofaciens* and *Bifidobacterium longum* having the highest LDA scores (LDA > 4.57; Figure 7) as compared with samples from diarrheic calves of the same age group.

Two weeks after experiencing diarrhea, 9 bacterial species were more abundant in fecal samples from calves on D2. Post-diarrheic calves exhibited higher abundance of *Collinsella aerofaciens* (LDA = 4.95) and 2 unidentified species (amplicon sequence variants, or ASV) of the *Blautia* genus (LDA > 4.46) as compared with healthy calves of the same age group. On the other hand, fecal samples from calves that were 4–5 weeks old and remained healthy throughout the study demonstrated 13 differently enriched species, including an unidentified species of the *Alloprevotella* genus (LDA > 4.05) and *Prevotella copri* (LDA = 4.16; Figure 8).

## 4 Discussion

During the first few weeks of life, diarrhea is the most common illness in dairy calves. As calves age, the risk of respiratory disease surpasses the risk of enteric disease (Hulbert and Moisá, 2016). With these patterns of disease progression in mind, this project aimed to describe the fecal microbiota of dairy calves from birth to 5 weeks old on 2 dairy farms before, during, and after GI disease as evidenced by diarrheal events uncomplicated by comorbidities. We hypothesized that microbial diversity, abundance, and composition would differ based on calf age and the presence or absence of GI disease progression.

The bacterial composition of the gut microbiota is important for the health and metabolism of adult dairy cows (Xu et al., 2021) and calves (Abe et al., 1995). Previous literature has demonstrated differences in the fecal microbiota of calves associated with breeds and clinical GI disease severity (Dorbek-Kolin et al., 2022; Slanzon et al., 2022), though limited information is available regarding aspects of the microbiome before and after the resolution of this disease. Therefore, exploring microbiome differences throughout the progression of uncomplicated GI disease in neonatal dairy calves provides baseline information for understanding GI disease microbial dynamics.

In this study, beta diversity analyses demonstrated differences in the fecal microbial composition of calves grouped by age and health status. However, the lack of separation between health status within an age group highlighted the dominant impact of age on the fecal microbiota composition of calves. Similarly, the general lack of separation between farms within an age group for healthy animals further substantiated the influence that age had on the calves’ fecal microbial community.

### 4.1 Bacterial diversity and differential abundance

Overall, differences in alpha and beta diversity measures of the fecal microbiome were predominantly associated with age-related changes as opposed to the calf health status. As the calves in our study aged, fecal taxa changed as well as increased in alpha diversity which is consistent with previous reports (Oikonomou et al., 2013; Dill-McFarland et al., 2017). In fact, pairwise comparisons showed no differences across health status (healthy vs. pre-, during, and post-diarrhea) within sampling times except for a difference between healthy and diarrheic calves 2–3 weeks old on D2. Age-related differences were expected given that fecal microbiome diversity has been reported to change rapidly throughout the early life of calves (Alipour et al., 2018), with microbiota profiles shown to differ between weeks of life throughout the preweaning period (Oikonomou et al., 2013). Insights into the progression of the calf microbiome from the neonatal period through weaning could help define what constitutes a healthy versus dysbiotic microbiome during growth and development. For example, a study that evaluated fecal samples from Holstein calves at 6 timepoints between 2 and 13 weeks of age demonstrated that bacterial richness, estimated by the number of observed species, and bacterial diversity, estimated by the Shannon diversity index, differed significantly between timepoints and increased over time (Hennessy et al., 2020). A more recent study that monitored the time-dependent dynamics of the gut microbiota of dairy calves before weaning, revealed that continuous changes including increasing gut microbiome diversity occurred from 1 to 5 weeks of age (Kim et al., 2021). In fact, the development of a robust microbiome is crucial to the health and development of dairy calves, necessary for a functioning immune system, and may provide some measure of protection against GI disorders (Gomez et al., 2019).

Even so, previous studies including our own have demonstrated differences in the fecal microbiome of calves with varying GI disease severity and associated treatments (Oikonomou et al., 2013; Gomez et al., 2017; Kim et al., 2021; Slanzon et al., 2022). Even though we only observed differences in diversity in the present study between healthy and diarrheic calves 2–3 weeks old on D2, there were specific bacteria that were differentially enriched when comparing calves’ health status and ages on both D1 and D2 based on LDA analysis (Figures 4–8). Many of these differences involved bacteria from the phyla Actinobacteria, Bacteroidetes, Firmicutes, and Proteobacteria that are frequently associated with calf GI disease of variable severity and progression, and that at the species-level raise interesting points for discussion. For example, *Bifidobacteriuam longum*, *Collinsella aerofaciens*, and *Eggerthella lenta* characterized the fecal microbiome of healthy calves 2–3 weeks old on D2, which agrees with our previous results from a study conducted at a different time and location (Slanzon et al., 2022). *Collinsella* produce ursodeoxycholic acid, which has been shown to reduce gene expression of proinflammatory cytokines IL-6 and TNF-α (He et al., 2022). Members of the genus *Bifidobacterium* in particular are abundant in the fecal microbiota of milk-consuming calves (Alipour et al., 2018), and may promote host health through immunomodulation, pathogen antagonism, maintenance of the intestinal mucosal barrier, and gut microbiota development among other beneficial GI-related effects (Hart et al., 2004; Vlkova et al., 2006; Lee and O'Sullivan, 2010; Fukuda et al., 2011; Aw and Fukuda, 2019). Similarly, *Bifidobacterium catenulatum* was enriched in healthy calves 2–3 weeks old on D1 and has been associated with increased butyrate levels in mice (Kim et al., 2020). Butyrate is considered beneficial as it has been shown to improve antioxidant function and growth of pre-weaned dairy calves (Liu et al., 2021; Rodríguez et al., 2022). *Bifidobacterium* strains capable of metabolizing non-digestible carbon sources (e.g., *B. catenulatum*) may support the carbohydrate availability of acetate-dependent butyrate producers by producing a high amount of acetate as an end-product in a nutrient-restricted environment (Moens et al., 2014). This suggests that the ability of bifidobacteria to degrade long-chain carbohydrates may affect the growth of other bacteria in co-culture. In fact, co-culture with *B. catenulatum* was recently shown to improve the growth, gut colonization, and butyrate production of acetate dependent *F. prausnitzii* (Kim et al., 2020).

In the current study *B. catenulatum* was identified as a biomarker in pre-diarrheic calves (0–1 week old) on D1. Furthermore, *Clostridium cadaveris, Clostridium paraputrificum, Clostridium perfringens* and *Escherichia coli* characterized the fecal microbiome of 0–1 week old healthy calves on D1. These findings are counterintuitive given the presumed alignment of *B. catenulatum* with a healthy status, and typical association of *C. paraputrificum*, *C. perfringens* and pathogenic *E. coli* strains with human and animal enteritis (Morris et al., 2011; Kiu et al., 2017). On the other hand, some strains of *C. cadaveris* have been shown to convert tryptophan to indolepropionic acid which has been shown to reduce intestinal permeability (Ma et al., 2018), and promote intestinal barrier function in mice (Venkatesh et al., 2014). This raises the point that conclusions based on individual species-level differences are difficult to interpret given the diverse and complex ecology of the GI microbiota across various health status. Perhaps microbial differences associated with variable health should be painted with broad brush strokes such as recent metagenomic and metabolomic analyses suggesting that gut microbiota-driven metabolic disorders of purine or arachidonic acid are associated with calf diarrhea (Shi et al., 2023). Ultimately, these various studies contribute to the conversation regarding the most appropriate mechanisms for modulating the GI microbiota to alleviate diarrhea in calves. Given that an altered gut microbiota plays a role in diarrhea pathogenesis, gut microbiota-targeted therapies could be useful for both prevention and treatment of diarrhea (Shi et al., 2023). In this study, changes in abundance related to health status were only observed for a few species, and significant shifts in the calf fecal microbial community during the onset of the disease were only observed on one dairy but not the other. These findings highlight not only the complexity of identifying when mechanisms for modulating the GI microbiota should be used, but also the challenges associated with developing microbial-based products that may require the inclusion of a complex combination of microbial species to effectively address different severities of GI disease and diverse farm-level impacts.

### 4.2 Case definitions

Nonetheless, diarrhea is simply a symptom that often serves as the diagnoses and sole identifier for a GI disease case definition. Numerous other factors such as age, nutritional and environmental variations, disease qualifiers such a pyrexia or inappetence, and treatment modalities ranging from oral electrolytes to antimicrobials inevitably play a role in determining the microbial response to GI disease. A recent scoping review of neonatal case definitions highlights the lack of clear case definitions for reporting neonatal calf diarrhea within the literature (Wilson et al., 2023). For example, after screening articles for inclusion within the review there were 54 studies that reported diarrhea outcomes based on treatment records, but only 29 of those used treatment records as part of the definition. Furthermore, 52 studies reported mortality related to diarrhea, but only 3 discussed deaths in the case definition for diarrhea. Even in studies with a defined case definition it was sometimes unclear if the definition was applied consistently for each diarrhea-related outcome.

For the purposes of the current study, we attempted to limit case definition variability by selecting diarrheic calves based on the presence of diarrhea (fecal score of ≥2) during 2–3 weeks old with an absence of clinical disease 2 weeks before and after the diarrheal event. Although we did not conduct fecal dry weights as part of this study, a previous study from our research group using the same descriptors of fecal consistency demonstrated that the average fecal dry weight (median and interquartile range, IQR) of samples from healthy calves (i.e., well-formed and semi-formed feces) was 27.8% (28.0%, IQR 9.1%) whereas samples from diarrheic calves (i.e., loose and watery feces) averaged 14.3% (13.1%, IQR 9.9%; Slanzon et al., 2022). As stated above, calves were excluded from analyses in the current study if they demonstrated abnormal behavioral scores outside of the designated diarrheal interval of 2–3 weeks of age. Furthermore, calves were excluded if they were pyrexic, had signs of respiratory disease, or were treated with laxatives, anti-inflammatories, or antimicrobials.

In conclusion, the purpose of this study was to evaluate fecal microbial changes before, during, and after uncomplicated GI disease in dairy calves as evidenced by diarrhea. By implementing a strict case definition, we were able to exclude factors that were not related to GI disease but could potentially alter the calf fecal microbial composition. Ultimately, this study highlights how shifts in the calf fecal microbial composition related to uncomplicated GI disease can differ across dairy farms, as evidenced by the differences in the fecal microbial composition between healthy and diarrheic calves (2–3 weeks old) observed in one dairy farm (D2) but not in the other (D1). Specific bacteria were identified as differently abundant in pre-, during, and post-diarrhea sampling points when compared with samples from healthy calves at the same age group; however, the changes in the calf fecal microbial composition were strongly correlated with age rather than health status or farm. This indicates that the age of the preweaned calf is the major driver of changes to fecal microbiome composition and diversity even with the presence of uncomplicated GI disease.

## Data availability statement

The fecal microbiome sequences have been deposited in the NCBI database under BioProject accession number PRJNA1041052 and under the Sequence Read Archive (SRA) accession IDs of SAMN38273304- SAMN38273561.

## Ethics statement

The animal studies were approved by The research protocol was reviewed and approved by the Institutional Animal Care and Use Committee of Washington State University (IACUC protocol #6859). The studies were conducted in accordance with the local legislation and institutional requirements. Written informed consent was obtained from the owners for the participation of their animals in this study.

## Author contributions

RC-W: Conceptualization, Data curation, Formal analysis, Investigation, Visualization, Writing – original draft, Writing – review & editing. GS: Conceptualization, Data curation, Formal analysis, Investigation, Software, Writing – original draft, Writing – review & editing. LE: Data curation, Writing – original draft, Writing – review & editing. HH: Data curation, Writing – original draft, Writing – review & editing. CMM: Data curation, Writing – original draft, Writing – review & editing. LP: Data curation, Project administration, Writing – original draft, Writing – review & editing. ST: Data curation, Writing – original draft, Writing – review & editing. CSM: Conceptualization, Data curation, Funding acquisition, Investigation, Methodology, Project administration, Resources, Supervision, Writing – original draft, Writing – review & editing.
