# Supplementary material for: Characterization of the preweaned Holstein calf fecal microbiota prior to, during, and following resolution of uncomplicated gastrointestinal disease
Source: Front Microbiol. 2024 May 24;15:1388489. doi: 10.3389/fmicb.2024.1388489 (PMC11157069; doi:10.3389/fmicb.2024.1388489)
Supplement: Supplementary file 5 [file Table_4.DOCX]

Supplementary Material

Characterization of the preweaned Holstein calf fecal microbiota prior to, during, and following resolution of uncomplicated gastrointestinal disease

Rachel A. Claus-Walker^1^†, Giovana S. Slanzon^2^†^*^, Lily A. Elder^1^, Holly R. Hinnant^1^, Chris M. Mandella^1^, Lindsay M. Parrish^1^, Sophie C. Trombetta^1^, Craig S. McConnel^1^

^1^Department of Veterinary Clinical Sciences, Field Disease Investigation Unit, Washington State University, Pullman, Washington, United States

^2^Current address: Department of Tropical Plant and Soil Sciences, University of Hawai'i at Mānoa, Honolulu, Hawaii, United States

†These authors contributed equally to this work and share first authorship

*** Correspondence:**Giovana S. Slanzon
giovanas@hawaii.edu

# Supplementary Figures and Tables

## Supplementary Figures

**Supplementary Figure 1.** Mean relative abundance of the most abundant ASVs across all samples. Relative abundance data was calculated based on the number of sequences reads and total reads per sample average across groups (health status, age, and farm). Different colors represent different ASVs.

# 1.2 Supplementary Tables

**Supplementary Table 1.** Behavioral score assessments of demeanor, ear position, mobility, interaction, suckling reflex, scleral injection, and ocular recession.

**Supplementary Table 2.** Summary of each calf health status based on clinical and behavior assessments.

**Supplementary Table 3.** ASV abundance table, taxonomy assignment table, and read processing summary table.
